# Experimental Research and Optimization of Ti-6Al-4V Alloy Microgroove Machining Based on Waterjet-Guided High-Power Laser

**DOI:** 10.3390/ma15217430

**Published:** 2022-10-23

**Authors:** Qian Liu, Yugang Zhao, Jianbing Meng, Zhilong Zheng, Chen Cao, Guoyong Zhao, Chuang Zhao, Guangxin Liu, Di Dai, Zhuang Song

**Affiliations:** School of Mechanical Engineering, Shandong University of Technology, Zibo 255049, China

**Keywords:** waterjet-guided laser, multifocus coupling, microgroove, RSM, process optimization

## Abstract

In order to improve the tribological properties of Ti-6Al-4V alloy and further broaden the application scope of titanium alloy materials in the industrial field, a preparation method of a waterjet-guided high-power laser processing surface microgroove was studied. In this paper, a multifocus coupling lens was innovatively designed to replace the spherical lens in the traditional waterjet-guided laser coupling device, which avoids the gas explosion phenomenon in the coupling of the high-power laser and waterjet, and realizes the high-quality coupling of the high-power laser and water beam fiber. Then, with the microgroove morphology as the response target, the single-factor test and response surface test of the water-guided laser processing microgroove were carried out. Based on the experimental results, an approximate mathematical model of the response surface between the process parameters and the microgroove topography target was constructed, and the quantitative relationship between the waterjet-guided laser processing parameters and the target response was studied. At the same time, the optimal combination of process parameters was obtained by multiobjective optimization, so as to effectively improve the microgroove morphology. This technology provides method guidance and a decision-making reference for subsequent waterjet-guided laser processing of titanium alloy surface functional microstructures.

## 1. Introduction

Titanium alloys have attracted wide attention in aerospace, biomedical, petrochemical, and other fields due to their high specific strength, corrosion resistance, high temperature resistance, and good biocompatibility, and are known as a “space metal” [1,2,3,4]. Ti-6Al-4V alloy is a two-phase α + β titanium alloy containing both an α-stable element (Al) and β-stable element (V). It has good comprehensive properties and is mainly used to manufacture aeroengine blades, artificial joints, cardiovascular stents, etc. [5,6]. However, low surface hardness, high friction coefficient, and poor wear resistance seriously hinder the application of Ti-6Al-4V alloy in some specific fields, so it is urgent to further improve the surface properties of titanium alloy [7,8]. The performance of titanium alloy in friction, wear, and corrosion depends on the surface of the material rather than the overall performance. Therefore, the application range of Ti-6Al-4V alloy can be further expanded by improving the properties of the material through surface modification on the premise of maintaining the bulk properties of the material.

Various surface modification methods have been successfully used to improve the tribological properties of Ti-6Al-4V titanium alloys, such as plasma spraying, thermal oxidation, laser cladding, surface texture, etc. [9,10,11,12,13,14,15]. Among them, surface texture is considered as an effective method to improve the tribological properties of materials [16]. The so-called surface texture refers to the use of a specific processing method to process an array of tiny pits, grooves, or other shapes with a certain size and arrangement on the surface of the workpiece. It is found that the microgroove texture has good abrasive collection ability, and the hard melting layer protrusion at the edge of the groove can effectively reduce the actual contact area of the friction pair surface, which makes the microgroove texture also have good antiwear ability under dry friction state [17].

At present, the high-energy laser beam processing method is widely used in the preparation and research of surface texture due to its simple process, high processing precision, and good controllability [18,19,20]. However, there are serious defects in traditional laser processing, such as the heat-affected zone, processing slag, and microcracks. In order to solve these problems, there are some processing methods that combine a laser with other processing methods, such as laser and electrolysis composite processing technology [21], laser and milling composite processing technology [22], and laser and waterjet composite processing technology [23]. Waterjet-guided laser processing technology is an advanced composite processing technology that combines traditional laser processing technology with waterjet processing technology. The principle of waterjet-guided laser processing is to import a high-energy laser into the high-speed flow of waterjet. After multiple total reflections of the laser on the inner surface of the waterjet, the water beam fiber with uniform cross-sectional energy distribution is formed. The water beam fiber has complex physical and chemical interactions with the workpiece material to achieve material removal [24]. Compared with the traditional laser-processing technology, waterjet-guided laser processing can not only overcome the problems of defocusing of the laser spot and the uneven distribution of laser energy, but also effectively reduce the heat-affected zone and hot slag on the machined surface [25].

At present, the research on the water-guided laser processing of microgrooves or cutting is mainly based on low-power nanosecond lasers. For example, Madhukar [26] proposed the fabrication of grooves on silicon’s surface without microcracks, a recast layer, and debris by using waterjet-assisted fiber laser technology, and studied the influence of different process parameters on the quality of grooves through a series of experiments. Adelmann [27] studied the effect of process parameters such as laser power on the depth of cut, and analyzed the maximum depth of cut for three different materials through experiments. Sun [28] verified the feasibility and characteristics of the waterjet-guided laser cutting of carbon-fiber-reinforced composites (CFRP), and found that compared with traditional laser processing, waterjet-guided laser processing has certain advantages for the HAZ and verticality of CFRP materials. However, the use of a nanosecond laser has the problems of low processing efficiency and high equipment maintenance cost. When the millisecond laser or even continuous laser is used, once the average power of the laser is too large, a gas explosion will occur when the laser exceeds the evaporation threshold of the water body, which will lead to the failure of water–laser coupling and even damage the nozzle element. Therefore, the realization of waterjet-guided long-pulse-width and high-power laser processing has always been a difficult problem.

This paper will carry out related research on the waterjet-guided high-power laser processing of Ti-6Al-4V alloy microgrooves. First, in order to avoid a gas explosion and other phenomena during waterjet-guided high-power laser processing, a new type of multifocusing point coupling lens was developed, thereby realizing high-quality coupling between a laser with an average power of 400 W and a water beam fiber. Then, taking the microgroove topography as the response target, single-factor experiments and response surface tests of the waterjet-guided laser processing microgrooves were carried out. Based on the experimental results, an approximate mathematical model of the response surface between the process parameters and the microgroove topography target was constructed, and the quantitative relationship between the waterjet-guided laser processing parameters and the target response was studied. At the same time, the optimal combination of process parameters was obtained by multiobjective optimization, so as to effectively improve the microgroove morphology. This technology provides method guidance and a decision-making reference for subsequent waterjet-guided laser processing of titanium alloy surface functional microstructures.

## 2. Equipment and Mechanism

### 2.1. Materials

The experimental samples were made of Ti-6Al-4V alloy sheets with dimensions of 50 mm × 50 mm × 2 mm. The main components and mechanical properties are shown in Table 1 and Table 2, respectively. Silicon carbide sandpapers of 800#, 1200#, 2000#, and 2500# were successively used to grind and polish the sample surface. The samples were cleaned with ethanol in an ultrasonic cleaner for 15 min, and then dried at room temperature.

### 2.2. Mechanism

In the process of waterjet-guided laser processing, the high-quality coupling between the laser and the water beam fiber is the key to realizing the waterjet-guided laser processing. When the laser pulse energy or continuous laser power is relatively small, the temperature of the water at the water–laser coupling position is limited and cannot reach the boiling-point temperature of the liquid. The water at the laser focus produces a temperature difference due to uneven heating, that is, a temperature gradient. Water expands differently at different temperatures, resulting in periodic changes in the density of water. However, at this time, the liquid water does not change its physical state and remains liquid. When the laser pulse energy density increases or the laser focus diameter decreases, the heated temperature at the coupling point of the laser and the waterjet exceeds the boiling point of the water and causes the water to vaporize; then, the vaporization will cause the liquid to boil or form bubbles to expand underwater and blast. This phenomenon can only be triggered by exceeding the vaporization energy, so this phenomenon has a threshold effect. In order to avoid the phenomenon of liquid boiling or bubble explosion when the high-power laser is coupled with the water beam fiber, the quality of the water beam fiber formed by the coupling of the laser and the waterjet is guaranteed. A method of “using an aspherical multifocusing point lens instead of a spherical single-focusing point lens to reduce the energy density of the focused spot at the water–laser coupling point and avoid gas explosion” is proposed to realize waterjet-guided high-power laser processing.

**Figure 1:**
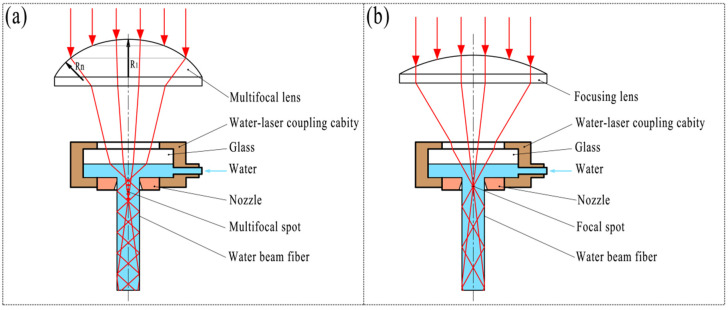
Schematic diagram of waterjet-guided laser coupling principle. (**a**) Multifocus coupling; (**b**) Single-focus coupling.

The principle of water–laser coupling in the waterjet-guided laser processing technology is shown in Figure 1. After the laser passes through the multi-focal-point lens, multiple focal points are formed at the mesoscopic scale along the axis of the water beam fiber. The laser is focused on each spot and then diverges, and is transmitted along the waterjet in the form of total reflection to realize water–laser coupling. At this time, the laser energy is evenly distributed in the formed multiple focal points, and the energy density of each focal point is significantly reduced, which effectively avoids the occurrence of phenomena such as boiling and bubble explosion in the waterjet. After a series of experiments, it has been proved that the coupling method can withstand more than 400 W of laser power.

**Figure 2:**
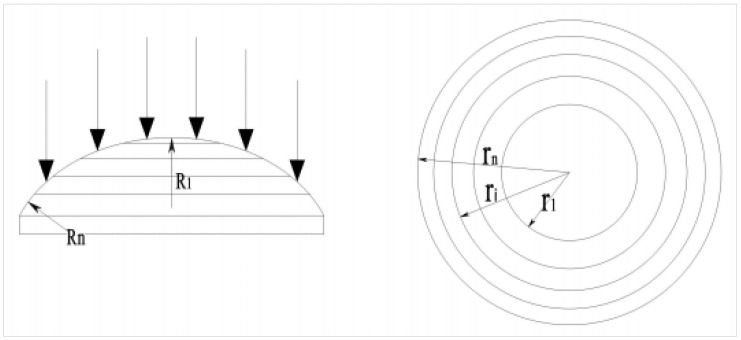
Schematic diagram of multifocus lens.

The multifocus lens is formed by the intersection of multiple spherical surfaces with the same axis and different radii. The annular spherical surface at the edge of the lens has the smallest radius *R_n_*, and the focal point formed by the laser passing through the annular spherical lens is closest to the bottom surface of the lens. The spherical surface at the top of the lens has the largest radius *R*_1_, the focal point formed by the laser passing through the spherical surface is the farthest from the bottom surface of the lens, and finally, *n* focal points uniformly distributed along the axis of the lens are formed, as shown in Figure 2.

The projected area of the top spherical surface of the multi-focal-point lens and each annular spherical surface along the axis line is equal, so that the laser energy density of each focus point in the waterjet is almost equal. Let the total number of top spherical surfaces and annular spherical surfaces of the multi-focal-point lens be *n*; the radius of the plane circle projected by the top spherical surface on the horizontal plane along the axis line is *r*_1_, and the outer circle radius of the plane ring projected by the outermost annular spherical surface on the horizontal plane along the axis line is *r_n_*, as shown in Figure 2. In the case of known *r_n_*, the calculation formula between the radius of the *i* plane circle and the outer circle of the plane ring is:

(1) $$r_{i}=\sqrt{\frac{1}{n}\times r_{n}^{2}}$$

where *r_i_* is the chord length of each curvature of the multi-focal-point lens. *n* is the total number of spherical and annular spherical surfaces at the top of the multi-focal-point lens.

In this paper, the target is a 5-focal-point lens, that is, *n* = 5. The radius *r*_5_ of the outer circle is consistent with the radius of the incident parallel light. When the radius of the parallel light is 8 mm, the plane circle sizes of the multifocus lens can be obtained from the above formula: *r*_1_ = 3.578 mm, *r*_2_ = 5.060 mm, *r*_3_ = 6.197 mm, and *r*_4_ = 7.155 mm. The curvature radius of the multifocusing lens can be obtained by the formula *R* = *f*(η − 1), where *f* is the focal length of the lens and *η* is the refractive index of the lens.

### 2.3. Equipment

**Figure 3:**
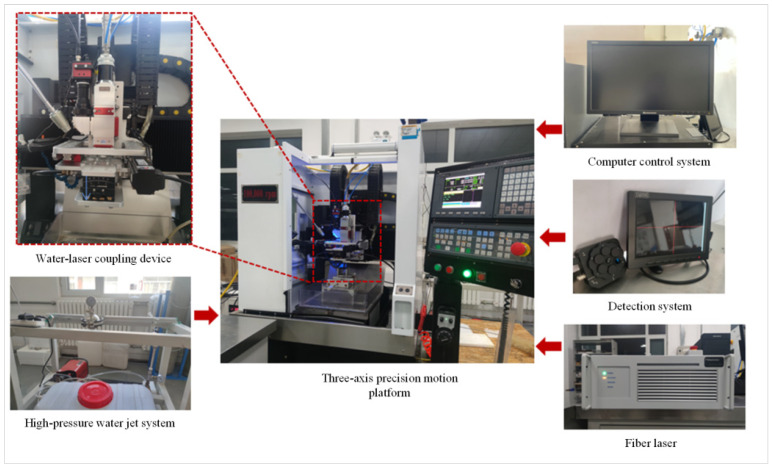
High-power waterjet-guided laser processing system.

The high-power waterjet-guided laser processing system obtained by the coupling of the multifocus laser and waterjet is shown in Figure 3. The system is mainly composed of a fiber laser (YLR-2000-WC, IPG), a water–laser coupling device, a computer control system, a high-pressure waterjet system, and a three-axis precision motion platform. The maximum average power of the fiber laser is 2000 W, the pulse frequency is adjustable in the range of 10 Hz~50 kHz, and the minimum pulse width is 20 µs. The processing range of the *X*-axis and *Y*-axis table of the equipment is 300 mm × 300 mm, the repeated positioning accuracy of the *Z*-axis of the water–laser coupling device is ±1 μm, and the maximum speed of the table movement is 1000 mm/s. Table 3 lists the main technical parameters of the waterjet-guided laser processing system. The design and manufacture of the high-power waterjet-guided laser processing system conforms to international standards, relevant safety requirements, and Chinese environmental protection standards. The geometric accuracy, working accuracy, and tolerance of the waterjet-guided laser processing device all meet the relevant standards. The measurement units of each component and various instruments adopt national standards.

After the experiment of waterjet-guided laser processing of the Ti-6Al-4V alloy microgrooves was completed, a 3D digital microscope (DSX1000, OLYMPUS) was used to observe the surface morphology of the microgrooves and measure the surface roughness of the sidewalls of the microgrooves. Analytical balance (Balance XSR205DU, METTLER TOLEDO) was used to measure the quality changes in samples before and after microgroove machining.

## 3. Design of Experiment

### 3.1. Single-Factor Experiments

**Figure 4:**
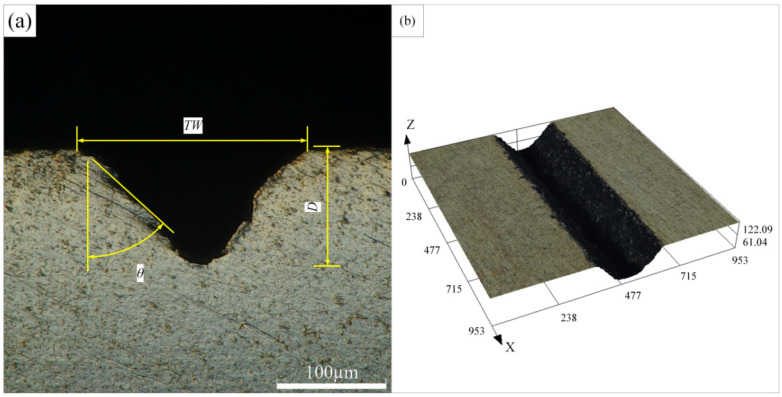
Schematic diagram of surface morphology of Ti-6Al-4V alloy microgroove. (**a**) Cross section of microgroove, (**b**) Three dimensional morphology of microgroove.

Figure 4 shows the surface morphology and cross-sectional profile of the Ti-6Al-4V alloy microgroove. The microgroove was formed after processing once at a laser power of 200 W, a laser pulse width of 50 μs, a pulse frequency of 5000 Hz, a cutting speed of 0.5 mm/s, and a water pressure of 1.8 MPa. It can be seen from the figure that the cross-section of the titanium alloy microgrooves after the waterjet-guided laser processing presents an obvious “V” shape. The reasons mainly include two aspects. First, when the focused laser is coupled with the coaxial waterjet to form a water beam fiber, in the case of near-center coupling, the energy density in the central region of the water beam fiber is higher than that in the surrounding region. The molten pool formed on the surface of the material in the central area of the water beam fiber is deep, and the molten pool formed in the surrounding area is shallow. The molten metal instantly forms a molten material under the cooling action of the waterjet and is washed away, forming a “V”-shaped microgroove cross-section with a wide upper and a narrow lower. Second, according to fluid mechanics, it can be known that the static pressure at the center of the waterjet is the largest, and the static pressure gradually decreases from the center to the outside. When the waterjet acts vertically on the surface of the workpiece, the molten material at the center of the waterjet will be eroded and discharged under the larger pressure of the waterjet. As a result, the depth of the microgroove at the center of the waterjet is the largest, forming a “V”-shaped microgroove cross-section.

The microgroove depth, microgroove top width, taper angle, and cross-sectional area can also be clearly seen from the figure. At the same time, it is found that there is no obvious heat-affected zone around the microgroove. The depth of the microgroove is about 109.275 µm, the top width of the microgroove is about 213.132 µm, and the taper angle is 47.7°. For waterjet-guided laser processing of the microgroove, it is expected to obtain a larger aspect ratio and material removal rate under the same waterjet-guided laser processing parameters. Therefore, the machinability evaluation indicators in this paper mainly include the depth of the microgroove, the top width of the microgroove, and the material removal rate. The material removal rate is calculated by dividing the workpiece mass loss by the processing time, which can be expressed as:

(2) $$MRR=\frac{M_{b}-M_{a}}{t}$$

where *M_b_* is the quality of the workpiece before processing, *M_a_* is the quality of the workpiece after processing, and *t* is the processing time.

**Figure 5:**
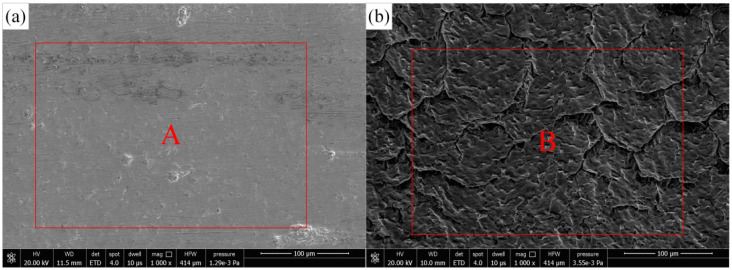
SEM of Ablation Surface of Microgroove and Original Surface of Substrate. (**a**) SEM of the original surface of the sample, (**b**) SEM of sample microgroove surface.

**Figure 6:**
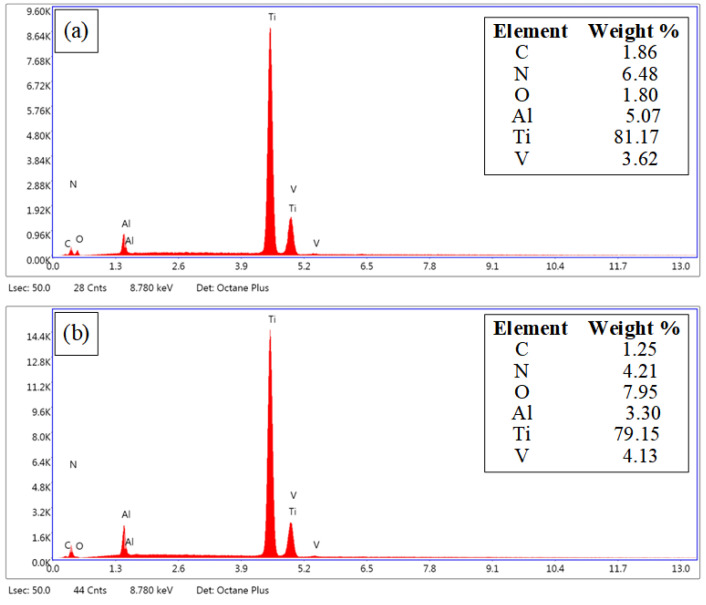
EDS of Ablation Surface of Microgroove and Original Surface of Substrate. (**a**) EDS of the original surface of the sample, (**b**) EDS of sample microgroove surface.

Figure 5 is the micromorphology of the original substrate surface and the microgroove ablation area obtained by the field emission scanning electron microscope (Apreo, FEI). Figure 6 shows the results of chemical composition content on the surface of the Ti-6Al-4V alloy original substrate and microgroove ablation area using an X-ray energy dispersive spectrometer (EDS) attached to an SEM. Figure 5a shows the original surface morphology of the Ti-6Al-4V alloy. The mass fraction of oxygen measured at the marker point A is 1.80%. Figure 5b shows the surface morphology of the microgroove ablation area. The mass fraction of oxygen measured at the marker point B is 7.95%. It can be seen from the EDS data that the oxygen content before and after processing is significantly increased, indicating that thermal ablation occurs during the processing of microgrooves, resulting in oxidation of the substrate by combining with oxygen in air or water.

A single-factor experiment refers to an experiment in which there is only one influencing factor in the experiment, or when there are multiple influencing factors and only one factor with the greatest influence is considered, and other factors are kept unchanged. In this paper, single-factor experiments were used to study the influence of waterjet-guided laser processing parameters on the surface morphology and surface quality of microgrooves. Table 4 shows the single-factor experiment scheme, which comprises 20 groups of experiments, each of which is repeated three times. The single-factor experiment’s goal is to find a suitable level for the response surface experiment.

### 3.2. Response Surface Methodology Experiments

Design of experiments (DOE) is the systematic method which dispenses the correlation between input and output responses. By utilizing the DOE, the experimental result is collected for validation and it is analyzed with a statistical method. In the present work, we adopt the response surface method (RSM) for experimental design and analysis. The RSM is a statistical method for solving multivariate problems by using a reasonable experimental design method. The experimental results are used as response values for data fitting and regression analysis [29,30]. The advantage of the RSM is that it can not only minimize the number of experiments and the level of independent variables, and save the cost of experiments, but also can evaluate the interaction between various factors and point out the nonlinear relationship between each factor and the response, which is conducive to optimizing the response.

The process of waterjet-guided laser processing is affected by a variety of process parameters, including average laser power, laser pulse frequency, laser pulse width, water pressure, water beam diameter, cutting speed, and cutting times. In order to investigate the effect of process parameters on the microgroove morphology, the RSM was used to design the experiment and then analyze the results. On the basis of previous experiments, we selected laser power, cutting speed, laser pulse width, and water pressure as the experimental design variables, and used microgroove depth, microgroove top width, and material removal rate as the response indicators. Except for the above four factors, the level values of other process parameters were fixed (the water beam diameter was 0.3 mm, and the laser pulse frequency was 5000 Hz), and the microgrooves were repeatedly cut four times to obtain a relatively large depth.

Usually, the experimental design of response surface analysis is divided into two types: central composite design (CCD) and Box–Behnken design (BBD). CCD generally adds a median value to the two-level test to evaluate the nonlinear relationship between output variables, action indicators, and factors. The BBD is optimal for two to five variables, and because there is no axial point in the BBD, it requires fewer test numbers with the same number of factors as the CCD [31]. Therefore, the BBD was used to determine the sample points of the response surface model, and the four variables were analyzed at three different levels. +1, 0, and −1 represented the high, medium, and low levels of each factor. Four factors and three levels of response surface tests were arranged, and a total of 29 groups of tests were arranged, including five groups of repeated tests at the same factor level, so as to facilitate the estimation of the main effect and interaction of each factor, reduce the relative error of the test, and avoid the test contingency. The experimental factors and level design are shown in Table 5.

In order to facilitate the expression of the mathematical model of the response later, *X*_1_, *X*_2_, *X*_3_, and *X*_4_ are used to represent the laser power, cutting speed, laser pulse width, and water pressure, respectively. The microgroove depth, microgroove top width, and material removal rate are denoted by *D*, *TW*, and MRR, respectively. The experimental scheme and experimental results based on the BBD are shown in Table 6.

## 4. Results and Discussion

### 4.1. Analysis of Single-Factor Experimental Results

#### 4.1.1. The Effect of Laser Power on the Microgroove Morphology

**Figure 7:**
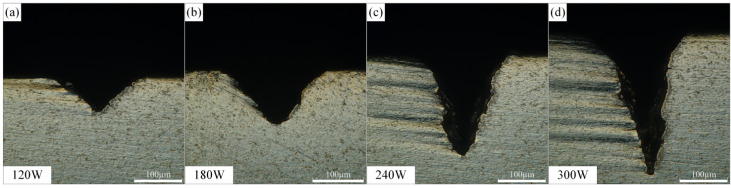
Effect of laser power on the microgroove morphology. (**a**) Laser power is 120 W, (**b**) Laser power is 180 W, (**c**) Laser power is 240 W, (**d**) Laser power is 300 W.

The effect of laser power on the morphology of Ti-6Al-4V alloy microgrooves during waterjet-guided laser processing is shown in Figure 7. According to the calculation of the single-pulse energy density, it can be known that when the average power of the waterjet-guided laser increased, the single-pulse energy density increased, the energy per unit area irradiated on the material was more, and it was easier to reach the damage threshold of the material. Over time, more material was removed through ablation. It can be seen from the figure that with the increase in laser power, the depth and cross-sectional area of the microgroove increased significantly, and when the laser power reached 300 W, the depth of the microgroove reached 294.658 µm, and the cross-sectional area reached 23,345.095 µm^2^. The effect of laser power on the microgroove top width was relatively insignificant. With the increase in laser power, the microgroove top width increased from 182.362 µm to 210.685 µm. It was found from the bottom of the microgroove that when the microgroove reached a certain depth, the laser energy gradually decreased with the increase in the depth of the microgroove. At the same time, due to the relatively narrowed outlet of the microgroove, a large amount of melt that could not be discharged in time appeared at the bottom of the microgroove, which reduced the actual depth of the microgroove.

#### 4.1.2. The Effect of Cutting Speed on the Microgroove Morphology

**Figure 8:**
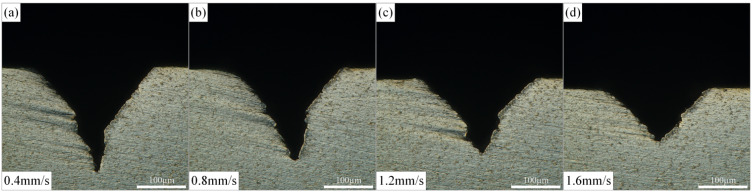
Effect of cutting speed on microgroove morphology. (**a**) Cutting speed is 0.4 mm/s, (**b**) Cutting speed is 0.8 mm/s, (**c**) Cutting speed is 1.2 mm/s, (**d**) Cutting speed is 1.6 mm/s.

The effect of cutting speed on the morphology of Ti-6Al-4V alloy microgrooves during the waterjet-guided laser processing is shown in Figure 8. From the calculation formula of the spot overlap ratio, it can be known that the spot overlap ratio was inversely proportional to the cutting speed when the diameter of the water beam fiber was consistent with the laser pulse frequency. As the cutting speed increased, the spot overlap ratio decreased, and within the same pulse duration, the energy absorbed by the material decreased, resulting in less material ablation. The results show that with the increase in cutting speed, the depth and cross-sectional area of the microgrooves decreased in turn. The depth difference of the microgrooves with 0.4 mm/s and 1.6 mm/s cutting speed was 103.859 μm, the cross-sectional area difference was 9578.769 μm^2^, and the decline rates were 49.2% and 47.8%, respectively. It can be seen that the cutting speed had a great influence on the morphology of the microgrooves. Compared with the influence of laser power on the top width of the microgroove, the effect of the cutting speed on the microgroove top width was relatively large. With the increase in cutting speed, the width of the microgroove top decreased from 256.427 µm to 217.162 µm.

#### 4.1.3. The Effect of Laser Pulse Width on the Microgroove Morphology

**Figure 9:**
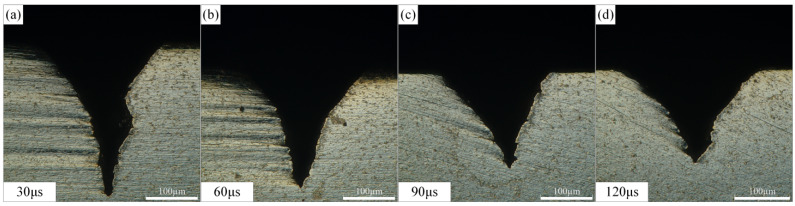
Effect of laser pulse width on microgroove morphology. (**a**) Laser pulse is 30 µs, (**b**) Laser pulse is 60 µs, (**c**) Laser pulse is 90 µs, (**d**) Laser pulse is 120 µs.

The effect of laser pulse width on the morphology of Ti-6Al-4V microgrooves during waterjet-guided laser processing is shown in Figure 9. When the average laser power and the laser pulse frequency are constant, according to the calculation formula of the laser peak power, the smaller the laser pulse width and the greater the laser peak power. When a laser with a short pulse width and high peak power interacts with the material, the full energy can be injected into a very small area at an extremely fast speed, and the high energy density in an instant will realize the removal of the material. It can be seen from the figure that as the laser pulse width increased, the depth of the microgrooves decreased. When the laser pulse width increased from 30 µs to 120 µs, the depth of the microgroove decreased from 290.683 µm to 166.493 µm, and the cross-sectional area decreased from 23,961.357 µm^2^ to 16,929.370 µm^2^. The laser pulse width had no significant effect on the groove top width, and its value was stable at around 260 µm (257.394 µm~260.873 µm).

#### 4.1.4. The Effect of Water Pressure on the Microgroove Morphology

**Figure 10:**
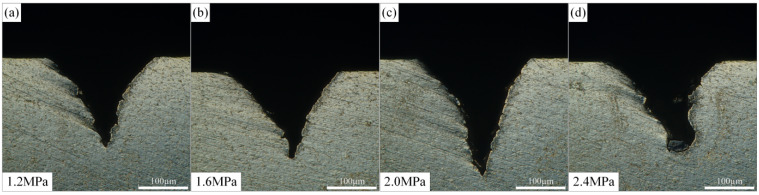
Effect of water pressure on microgroove morphology. (**a**) Water pressure is 1.2 MPa, (**b**) Water pressure is 1.6 MPa, (**c**) Water pressure is 2.0 MPa,(**d**) Water pressure is 2.4 MPa.

The effect of water beam pressure on the morphology of Ti-6Al-4V microgrooves during waterjet-guided laser processing is shown in Figure 10. The water beam fiber is the transmission medium of laser energy. At the same time, the waterjet has the effect of erosion and cooling on the workpiece, and has a significant inhibitory effect on the heat-affected zone generated during the processing. It can be found from the figure that with the increase in the water pressure, the depth and cross-sectional area of the microgrooves showed a trend of increasing first and then decreasing. This phenomenon is because as the pressure of the waterjet increased, the impact force of the waterjet increased, the erosion effect was enhanced, and the molten material on the surface of the material was removed under the erosion action of the waterjet. When the water pressure was too large, the stability of the water beam fiber was affected, resulting in the loss of laser power, thus affecting the processing effect. The top width of the microgroove was not sensitive to the change in the water pressure, and its value was stable at about 240 µm.

### 4.2. Analysis of Response Surface Experiment Results

#### 4.2.1. Regression Model and ANOVA

According to the adaptability analysis, the quadratic model is the most suitable model for the three responses of microgroove depth, microgroove top width, and MRR. Therefore, this paper used a multiple quadratic regression model to fit the functional relationship between the factor and the response value, and finally realized the optimization of the variable and the prediction of the response value. The quadratic regression model can be expressed as [32]:

(3) $$Y=\beta_{0}+\sum_{i=1}^{k}\beta_{i}x_{i}+\sum_{i=1}^{k}\beta_{ii}x_{ii}^{2}+\sum_{i=1}^{k}\sum_{j=1}^{k}\beta_{ij}x_{i}x_{j}+\varepsilon$$

where *Y* is the predicted response variable, and *i*,*j* are integers; *β_0_* and *β_j_* are regression coefficients; *β_ij_* represents the interaction effects of the different input variables *X_i_* and *X_j_*; and *β_jj_* represents the interaction of the input variable *X_i_* itself.

In this paper, the four-element quadratic regression equation was used to fit the microgroove depth, microgroove top width, and MRR, and some nonobvious items were deleted from the fitting results. The regression equation of the response is determined as follows:

(4) $$D=394.86+74.32X_{1}-13.83X_{2}-33.25X_{3}-2.01X_{4}-7.83X_{1}X_{3}+7.96X_{2}X_{3}+8.92X_{3}X_{4}+9.45X_{1}^{2}-9.25X_{3}^{2}-14.68X_{4}^{2}$$

(5) $$TW=230.99+14.5X_{1}-7.8X_{2}+0.3721X_{3}+0.6679X_{4}+8.77X_{1}X_{3}+4.31X_{1}^{2}-10.63X_{3}^{2}$$

(6) $$\begin{array}{l} MRR=12.42+3.05X_{1}+5.23X_{2}-0.9083X_{3}-0.0750X_{4}+1.35X_{1}X_{2}-0.3750X_{2}X_{4}\\ +0.6108X_{1}^{2}-0.4392X_{2}^{2}-0.8017X_{3}^{2}-0.6017X_{4}^{2} \end{array}$$

where *X*_1_, *X*_2_, *X*_3_, and *X*_4_ represent laser power, cutting speed, laser pulse width, and water pressure, respectively.

In order to further verify the accuracy of the model, the ANOVA method was used to determine the statistical parameters and fitting effect of the model, which is equivalent to identifying the key factors and stating which one is the most important factor. Additionally, factors such as R^2^, adjusted R^2^, predicted R^2^, and adeq precision (AP) were used to reflect the quality of the model [33]. Table 7 summarizes the important expressions for different metrics.

The results of the quadratic model ANOVA and *F* test of the depth of the Ti-6Al-4V alloy microgrooves processed by the waterjet-guided laser are shown in Table 8. The *F* value of the model selected in this experiment was 118.76, and the *p* value was less than 0.01, indicating that the response surface model with respect to the depth of the microgroove was very significant. When the *p* value of the model item was less than 0.05, it showed that the influence of the model item was very obvious. Therefore, *X*_1_, *X*_2_, *X*_3_, *X*_1×3_, *X*_2_*X*_3_, *X*_3_*X*_4_, *X*_1_^2^, *X*_3_^2^, and *X*_4_^2^ are all obvious model items in the microgroove depth model. In this study, the R^2^ of the microgroove depth model was 0.9917, and the Adjusted R^2^ was 0.9833. The adjusted R^2^ (0.9833) was very close to the predicted R^2^ (0.9569), and the AP value was 41.6304, indicating that the model had high regression. Therefore, this model can be used to analyze and predict the depth of microgrooves on the surface of Ti-6Al-4V alloy.

**Figure 11:**
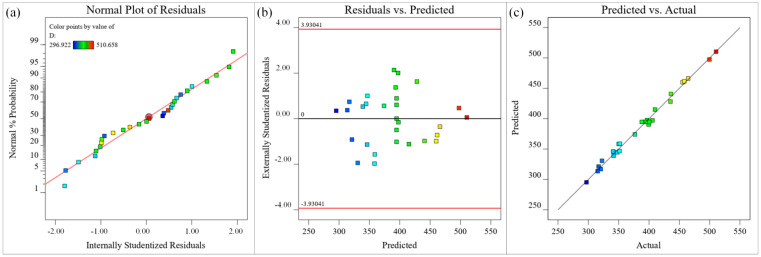
Diagnostic plots for depth (μm) (**a**) Normal plot of residuals, (**b**) Residuals versus predicted plot, (**c**) Predicted versus actual values plot.

The fit of the model can be assessed with several diagnostic plots, including the normal plot of residuals, the residuals versus predicted plot, and the predicted versus actual values plot. The different-colored data points in Figure 11 represent the microgroove depths under different process parameters. The residual normal probability of all data points in Figure 11a follows a straight-line distribution, indicating that the residuals follow a normal distribution and the microgroove depth model has good adaptability. The upper and lower lines in Figure 11b represent the distribution range of residuals. In the absence of constant error, the residuals of all microgroove depth predictions are randomly distributed within ±2.5 of the near-zero axis, which indicates that there is no obvious regularity among the residuals of microgroove depths. In Figure 11c, a small number of the data points are distributed on the straight line (y = x), and most of the data points are distributed around the straight line (y = x), indicating that the experimental value of the microgroove depth is in good agreement with the predicted value and the simplified quadratic. The model is suitable and acceptable for the experimental data. In summary, the obtained approximate model of microgroove depth has high significance, good adaptability, high reliability, and high prediction accuracy, and can accurately describe the nonlinear relationship between process parameters and microgroove depth.

The results of the quadratic model ANOVA and *F* test of the microgroove top width of the Ti-6Al-4V microgroove processed by the waterjet-guided laser are shown in Table 9. The *F* value of the selected model in this experiment was 27.56, and the *p* value was less than 0.01, indicating that the response surface model on the microgroove top width was very obvious. When the *p* value of the model item was less than 0.05, it indicated that the effect of the model item was very significant. Therefore, *X*_1_, *X*_2_, *X*_1_*X*_3_, *X*_1_^2^, and *X*_3_^2^ in the microgroove top width model were all significant model terms of the microgroove top width model. The R^2^ of the microgroove top width model was 0.9650 and the adjusted R^2^ was 0.9300. The difference between adjusted R^2^ (0.9300) and predicted R^2^ (0.8322) was small, and the AP value was 22.0022. It was proved that the model had the ability to predict the top width of the microgroove on the Ti-6Al-4V surface.

**Figure 12:**
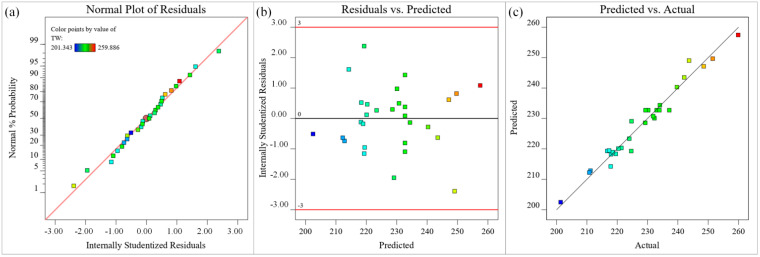
Diagnostic plots for top width (μm) (**a**) Normal plot of residuals, (**b**) Residuals versus predicted plot, (**c**) Predicted versus actual values plot.

The data points in different colors in Figure 12 represent the microgroove top width under different process parameters. The residual normal probability of each data point in Figure 12a is approximately linearly distributed, indicating that all data points are normal, and the adaptability of the microgroove top width model is good. The upper and lower lines in Figure 12b represent the distribution range of the residuals. The residuals of the predicted value of the microgroove top width are irregularly distributed near the zero axis within the distribution range, indicating that there is no obvious relationship between the residuals, and the randomness is good. In Figure 12c, a small number of the data points are distributed on the straight line (y = x), and most of the data points are distributed around the straight line (y = x), indicating that the experimental value of the microgroove top width is basically consistent with the predicted value, and the model fitting accuracy is relatively high. In summary, the regression fitting approximate mathematical model of the microgroove top width has high visibility, good adaptability, high reliability, and high fitting accuracy, which can accurately reflect the nonlinear relationship between process parameters and microgroove top width.

The results of the quadratic model ANOVA and the *F* test of the MRR of the Ti-6Al-4V microgrooves processed by the water-jet guided laser are shown in Table 10. The *F* value of the model selected in this experiment is 337.43, and the *p* value is less than 0.01, indicating that the response surface model on the MRR is very significant. When the *p* value of the model item is less than 0.05, it shows that the influence of the model item is very obvious. Therefore, *X*_1_, *X*_2_, *X*_3_, *X*_1_*X*_2_, *X*_2_*X*_4_, *X*_1_^2^, *X*_2_^2^, *X*_3_^2^, and *X*_4_^2^ are all obvious model items in the MRR model. The R^2^ of the MRR model is 0.9970 and the adjusted R^2^ is 0.9941. The difference between adjusted R^2^ (0.9941) and predicted R^2^ (0.9868) is very small, and the AP value is 75.0136. Therefore, this model can be used to analyze and predict the MRR.

**Figure 13:**
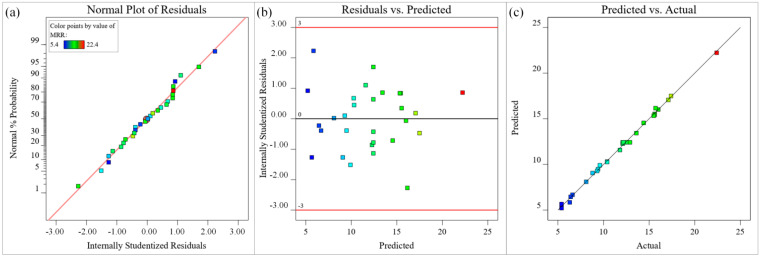
Diagnostic plots for MRR (μm): (**a**) Normal plot of residuals, (**b**) Residuals versus predicted plot, (**c**) Predicted versus actual values plot.

The different-colored sample points in Figure 13 represent the MRR under different process parameters. The normal probability of residuals of all sample points in Figure 13a is approximately a straight-line distribution, indicating that the residuals are normally distributed, and the MRR model has good adaptability. The upper and lower lines in Figure 13b represent the distribution range of residuals. The residuals of all predicted MRR are randomly distributed around the zero axis within the distribution range, which indicates that there is no obvious pattern between the residuals of MRR. Most of the data points in Figure 13c are distributed on the straight line (y = x), and a small part of the data points are distributed around the straight line (y = x), indicating that the experimental value of the MRR is in good agreement with the predicted value, and the model fitting accuracy is higher. In summary, the constructed approximate mathematical model for the MRR of titanium alloy microgrooves processed by a waterjet-guided laser has high adaptability and accuracy.

#### 4.2.2. Influence of Process Parameters on Response

**Figure 14:**
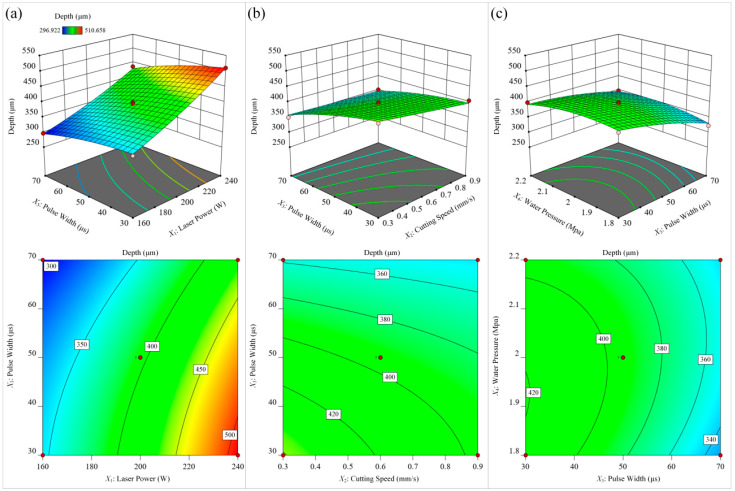
The influence of different process parameters on the depth. (**a**) laser power and pulse width; (**b**) cutting speed and pulse width; (**c**) pulse width and water pressure.

The 3D surface plot and contour plot of the response can directly reflect whether the interaction between the two factors has a significant effect on the response value. Figure 14 investigates the effect of different process parameters on the surface microgroove depth of Ti-6Al-4A alloy. Based on the significance of the items in the microgroove depth model, it can be seen that the laser power had the most significant effect on the microgroove depth, followed by the laser pulse width. Figure 14a shows that in the range of parameters, the highest laser power and the shortest laser pulse width reached the maximum depth, and the lowest laser power and the longest laser pulse width reached the minimum depth. The reason for this phenomenon may be that laser power is the main energy source in the process of waterjet-guided laser processing. The greater the laser power, the greater the laser energy density, and the greater the volume of the material surface to be melted. Under the impact of the waterjet, the molten metal was immediately discharged, resulting in deeper microgrooves. The smaller the laser pulse width, the greater the laser peak power, and the deeper the microgrooves formed on the surface of the material. It can be seen from Figure 14b that when the laser power and water pressure remained unchanged, as the cutting speed decreased, the laser energy transmitted from the water beam fiber to the material also increased, and the melted volume of the material surface increased, resulting in an increase in the depth of the microgrooves. According to Figure 14c, when the laser power and feed rate were constant, the pulse width was the shortest and the water beam pressure was around 2.0 MPa, the microgroove depth was the largest. Because the water pressure was small, the erosion force of the waterjet could not discharge the molten metal in the microgroove, resulting in a shallow microgroove. When the water pressure was too high, the stability of the water beam fiber decreased, and the energy transfer efficiency of the water beam fiber decreased, so the depth of the microgroove was reduced.

**Figure 15:**
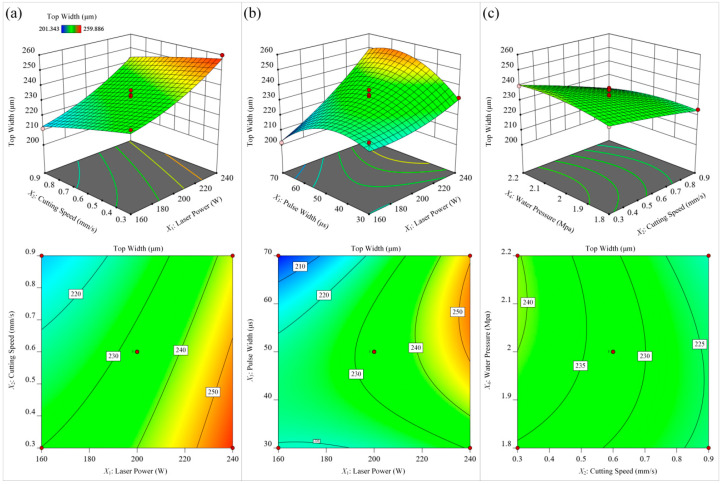
The influence of different process parameters on the width. (**a**) laser power and cutting speed; (**b**) laser power and pulse width; (**c**) cutting speed and water pressure.

Figure 15 investigates the effect of different process parameters on the microgroove top width of Ti-6Al-4V alloy. Based on the significance of the items in the microgroove top width model, it can be seen that the laser power had the most significant effect on the microgroove top width, followed by the cutting speed. As shown in Figure 15a, the microgroove top width was larger at a higher laser power and lower cutting speed. Conversely, when the laser power was decreased and the cutting speed was increased, the microgroove top width decreased. This is because the laser energy in the water beam fiber is approximately Gaussian distribution [34]; the energy in the central region of the water beam fiber is higher and the surrounding area is lower. Material removal is only achieved when the energy exceeds the material ablation threshold. With the increase in laser power, the energy transmitted by the water beam fiber also increased, and the area on the surface of the material that exceeded the ablation threshold also increased, resulting in a larger top width of the microgroove. As the cutting speed decreased, the laser energy absorbed by the material surface also increased, so the lower cutting speed increased the top width of the microgroove. As shown in Figure 15b, when the laser pulse frequency and water pressure were constant, the minimum microgroove top width of the microgroove was obtained under the condition of low laser power and long laser pulse width. Additionally, with the increase in laser power, the influence of laser pulse width on the microgroove top width changed from negative correlation to positive correlation. The reason is that the cooling effect of the waterjet is obvious when the laser power is low. At this time, the shorter laser pulse width had higher laser peak power, and the wider the width of the microgrooves formed on the surface of the material. As the laser power increased, the cooling effect of the waterjet decreased, and under the combination of high laser power and long laser pulse width, the energy transferred to the workpiece surface was very high, resulting in an increase in the top width of the microgroove. As shown in Figure 15c, the microgroove top width was larger at a lower cutting speed and higher waterjet pressure. However, when the water pressure continued to increase, the top width of the microgroove showed a decreasing trend. This is because a lower cutting speed increases the spot overlap, which results in more energy absorbed per unit area of material, which increases material removal and groove top width. At the same time, as the water pressure continues to increase, more heat is carried away by the water flow, thereby limiting the size of the ablated area and the top width of the microgroove.

**Figure 16:**
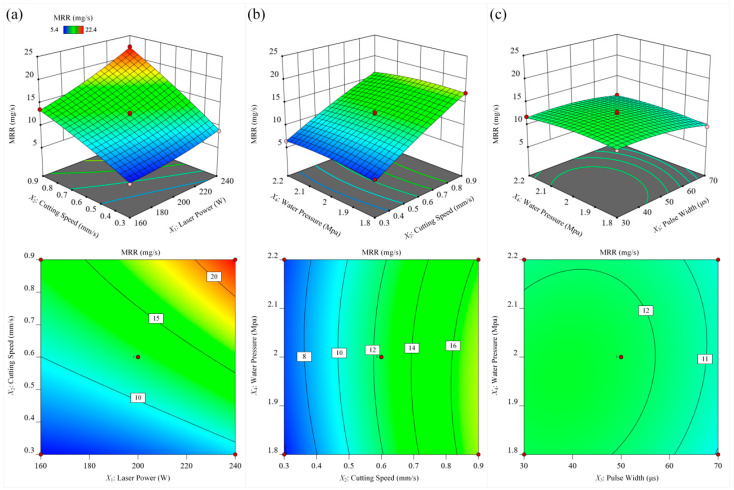
The influence of different process parameters on the MRR. (**a**) laser power and cutting speed; (**b**) cutting speed and water pressure, (**c**) pulse width and water pressure.

Figure 16 investigates the effect of different process parameters on the MRR. Based on the significance of the items in the material removal rate model, it can be seen that the cutting speed had the most significant effect on the MRR, followed by the laser power. It can be seen from Figure 16a that when the maximum laser power and the minimum cutting speed were combined, the MRR reached the maximum value. First, the increase in laser power transferred more laser energy to the workpiece, resulting in more material being removed. Second, as the cutting speed increased, the depth and width of the microgrooves decreased. However, due to the increase in cutting speed, the material removal amount per unit time increased. Therefore, the MRR increased with the increase in cutting speed within the test range. It can be seen from Figure 16b that when the laser power and laser pulse width were constant, the cutting speed had a significant effect on the MRR, while the waterjet pressure had no significant effect on the MRR. As the cutting speed increased, the MRR gradually increased. It can be seen from Figure 16c that when the cutting speed, laser power, and laser frequency were constant, the MRR increased with the decrease in the laser pulse width. When the laser pulse width was less than 50µs, the effect of the laser pulse width on the material removal rate was less affected. When the water beam pressure was between 1.8 MPa and 2.1 MPa, the effect of water pressure on the MRR was not significant. When the water pressure was too large, the MRR began to show a downward trend because the water pressure affecting the stability of the water beam fiber at this time, resulting in the loss of laser energy and the reduction in material removal.

### 4.3. Parameter Optimization and Experimental Verification

**Figure 17:**
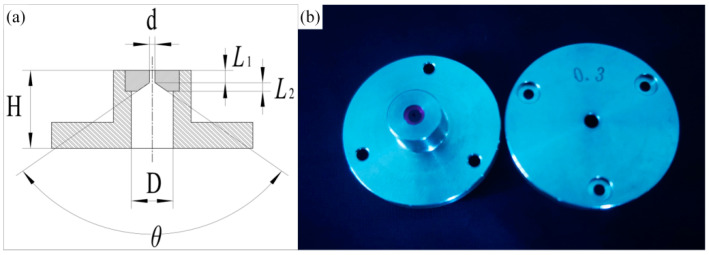
Nozzle model and object. (**a**) Nozzle model; (**b**) Picture of nozzle.

The diameter of the waterjet nozzle used in this paper was 0.3 m; the nozzle model and real object are shown in Figure 17 [35]. When the high-pressure waterjet is ejected from the nozzle hole, the phenomenon of shrinkage will occur. In fact, the diameter of the water beam fiber formed was about 83% of the nozzle hole diameter. At the same time, because the laser energy distribution in the water beam fiber is nearly Gaussian, the feature width of the microgroove top of the waterjet-guided laser processing microgroove was 230 µm. In order to obtain microgrooves with high aspect ratio, high processing efficiency, and excellent surface quality, the ratio of microgroove depth to microgroove top width was preset to 2; that is, the microgroove depth was about 460 µm. Then, the objective of the multiobjective optimization of the RSM was obtained as:

(7) $$\left\{ \begin{array}{l} MRR=\max\left\{MRR_{i}\right\},mg/s\\ TW=\left\{x|x\in \left[225,235\right]\right\},\mu m\\ D=\left\{y|y\in \left[450,470\right]\right\},\mu m \end{array}\right.$$

According to the quadratic model of each response and the limited range of optimization objectives, a series of optimal solutions were obtained. The three groups of optimization schemes for process parameters with the highest expected values are shown in Table 11.

**Figure 18:**
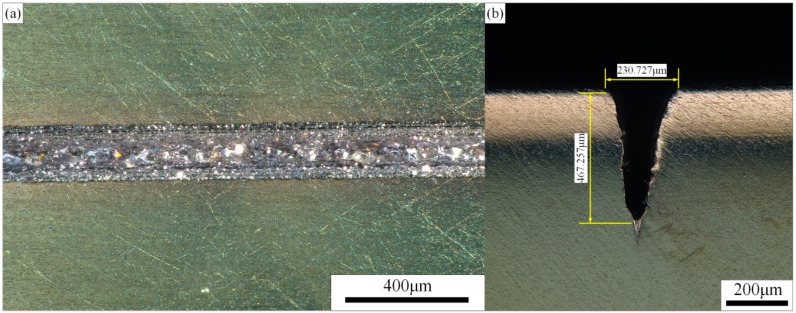
Microgroove morphology after optimization of process parameters. (**a**) Surface morphology, (**b**) Cross-sectional morphology.

In order to further verify the goodness of fit of the quadratic response surface model, validation tests were conducted on these three groups of optimization schemes within the technical parameters of the processing equipment. The results are shown in Table 12. The test results were measured with a 3D digital microscope and an analytical balance, and it was found that the comprehensive results of the second group of optimization schemes were closest to the target values. The titanium alloy microgroove morphology of the second optimization scheme is shown in Figure 18. The actual microgroove depth was 467.257 μm, the groove top width was 230.727 μm, and the MRR was 20.351 mg/s. The relative errors between the experimental and theoretical values of the corresponding processing parameters were 1.58%, 0.32%, and 6.47%, respectively.

The error may be caused by:

1. The instability and system error of the test equipment.
2. There is an error between the actual process parameters and the predicted process parameters.
3. The defect of the optimization method itself leads to the error.

The test results show that the error of the model is within the acceptable range, the model can better optimize the test parameters to achieve better test results, and the model can be used to predict the response.

## 5. Results and Discussion

In this paper, related research was carried out on the waterjet-guided high-power laser processing of Ti-6Al-4V alloy microgrooves. The following conclusions are drawn from the study.

1. This paper proposed a method of “using an aspheric multifocus lens instead of a spherical single-focus lens to reduce the energy density of the focused spot at the water–laser coupling point and avoid gas explosion disturbance”, thus realizing waterjet-guided high-power laser processing.
2. According to the results of the single-factor experiment, it was determined that the parameters such as laser power, cutting speed, and laser pulse width have a significant effect on the depth and top width of the microgroove, but have no significant effect on the HAZ and surface roughness of the microgroove. The water pressure has a significant effect on the stability of the water beam fiber, which in turn affects the transmission efficiency of the laser energy, thereby affecting the surface morphology of the microgroove.
3. In this paper, the quadratic regression model of laser power, cutting speed, laser pulse width, and water pressure on the index was established with the microgroove depth, microgroove top width, and MRR as the evaluation indexes, and the variance analysis was used to verify the good fitting effect. The 3D surface map and contour map reflect the influence law of different process parameters on the evaluation index and their interaction relationship.
4. In this paper, through multiobjective optimization, microgrooves with high aspect ratio, high processing efficiency, and excellent surface quality were obtained. The optimal process parameters are as follows: laser power is 238.6 W, cutting speed is 0.90 mm/s, laser pulse width is 36.8μs, and water pressure is 2.13 MPa. The Ti-6Al-4V alloy microgrooves with good morphology were fabricated by using the optimized process parameters. The actual depth, top width, and MRR of the microgrooves were 467.257 μm, 230.727 μm, and 20.351 mg/s, respectively. The prediction error was within the acceptable range.

## Figures and Tables

**Table 1:** Main chemical composition of Ti-6Al-4V alloy.

Element	Al	V	Fe	C	H	N	O	Ti
Mass Fraction/%	5.5~6.8	3.5~4.5	≤0.30	≤0.10	≤0.015	≤0.20	≤0.20	Bal.

**Table 2:** Mechanical properties of Ti-6Al-4V alloy.

Values	Hardness (GPa)	Modulus (GPa)	Tensile Strength (MPa)	Yield Stress (MPa)	Poisson Ration
Ti-6Al-4V	4.01	126	910	830	0.34

**Table 3:** Main technical parameters of the waterjet-guided laser processing system.

Parameter	Value
Wavelength/nm	1064
Laser power/W	≤2000
Pulse width/µs	≥50
Scan speed/(mm·s^−1^)	≤1000
Focal length/mm	98~100
Number of focus points	5
Water beam fiber diameter/mm	0.3

Parameter

Value

**Table 4:** Single-factor experiment scheme.

Serial Number	Laser Power (W)	Cutting Speed (mm/s)	Pulse Width (μs)	Water Pressure (MPa)	Processing Times
1	120	0.5	50	2.0	1
2	180	0.5	50	2.0	1
3	240	0.5	50	2.0	1
4	300	0.5	50	2.0	1
5	200	0.4	50	2.0	1
6	200	0.8	50	2.0	1
7	200	1.2	50	2.0	1
8	200	1.6	50	2.0	1
9	200	0.5	30	2.0	1
10	200	0.5	60	2.0	1
11	200	0.5	90	2.0	1
12	200	0.5	120	2.0	1
13	200	0.5	50	1.2	1
14	200	0.5	50	1.6	1
15	200	0.5	50	2.0	1
16	200	0.5	50	2.4	1
17	200	0.5	50	2.0	1
18	200	0.5	50	2.0	2
19	200	0.5	50	2.0	3
20	200	0.5	50	2.0	4

**Table 5:** Independent variable and experimental design level used in the BBD.

Variable	Symbol	Extreme Value		
		Low (−1)	Medium (0)	High (+1)
Laser Power (W)	*X*_1_	160	200	240
Cutting Speed (mm/s)	*X*_2_	0.3	0.6	0.9
Pulse Width (μs)	*X*_3_	30	50	70
Water Pressure (MPa)	*X*_4_	1.8	2.0	2.2

**Table 6:** Experimental design and results of the BBD.

Run Order	*X*_1_	*X*_2_	*X*_3_	*X*_4_	MRR (mg/s)	*D* (μm)	*TW* (μm)
1	160	0.3	50	2.0	5.4	341.623	232.274
2	240	0.3	50	2.0	8.8	499.695	259.886
3	160	0.9	50	2.0	13.6	316.901	211.186
4	240	0.9	50	2.0	22.4	455.483	242.086
5	200	0.6	30	1.8	12.1	409.911	217.966
6	200	0.6	70	1.8	9.6	322.478	219.521
7	200	0.6	30	2.2	11.8	399.371	218.548
8	200	0.6	70	2.2	10.4	347.602	221.361
9	160	0.6	50	1.8	9.4	320.464	216.727
10	240	0.6	50	1.8	15.6	464.499	248.521
11	160	0.6	50	2.2	9.3	315.317	217.329
12	240	0.6	50	2.2	15.6	458.182	251.495
13	200	0.3	30	2.0	6.6	436.257	224.727
14	200	0.9	30	2.0	17.4	405.663	210.814
15	200	0.3	70	2.0	5.4	350.044	229.245
16	200	0.9	70	2.0	15.5	351.291	217.857
17	160	0.6	30	2.0	10.4	340.881	224.623
18	240	0.6	30	2.0	16.0	510.658	231.867
19	160	0.6	70	2.0	8.1	296.922	201.343
20	240	0.6	70	2.0	14.4	435.397	243.683
21	200	0.3	50	1.8	6.3	399.399	234.069
22	200	0.9	50	1.8	17.1	376.795	223.973
23	200	0.3	50	2.2	6.4	397.005	239.683
24	200	0.9	50	2.2	15.7	351.944	220.376
25	200	0.6	50	2.0	12.3	391.432	232.994
26	200	0.6	50	2.0	12.1	388.172	230.220
27	200	0.6	50	2.0	12.6	398.671	233.905
28	200	0.6	50	2.0	12.9	400.473	237.165
29	200	0.6	50	2.0	12.2	394.724	229.329

**Table 7:** Expressions used for model verification.

Terms	Expressions	Remarks
$R^{2}$	$R^{2}=\frac{\sum_{i=1}^{n}{\left({\hat{y}}_{i}-\bar{y}\right)}^{2}}{\sum_{i=1}^{n}{\left(y_{i}-\bar{y}\right)}^{2}}=\frac{S_{Res}^{2}}{S_{T}^{2}}$	Close to 1.0 is ideal.
$R_{adjusted}^{2}$	$R_{adjusted}^{2}=1-\frac{S_{Res}^{2}/\left(n-p\right)}{S_{T}^{2}/\left(n-1\right)}$	Close to 1.0 is ideal.
*PRESS*	$PRESS={\sum_{i=1}^{n}\left(y_{i}-{\hat{y}}_{i}\right)}^{2}$	The value should be small.
$R_{predicted}^{2}$	$R_{predicted}^{2}=1-\frac{PRESS}{S_{T}^{2}}$	$\mathrm{No}\;\mathrm{more}\;\mathrm{than}\;0.2\;\mathrm{discrepancy}\;\mathrm{between}\;R_{adjusted}^{2}$$\;\mathrm{and}\;R_{predicted}^{2}$ should be expected.
Lack of Fit	$F_{LOF}=\frac{S_{LOF}^{2}/\left(f-p\right)}{S_{PE}^{2}/\left(n-p\right)}$	$F_{LOF}$ should be larger than 0.05.

**Table 8:** ANOVA of the regression model for depth.

Source	Sum of Squares	df	Mean Square	*F* Value	*p* Value
Model	85844.06	14	6131.72	118.76	<0.0001
Laser Power (W) *X*_1_	66273.82	1	66,273.82	1283.61	<0.0001
Cutting Speed (mm/s) *X*_2_	2294.84	1	2294.84	44.45	<0.0001
Pulse Width (μs) *X*_3_	13268.41	1	13,268.41	256.99	<0.0001
Water Pressure (MPa) *X*_4_	48.50	1	48.50	0.9394	0.3489
*X*_1_ *X*_2_	94.97	1	94.97	1.84	0.1965
*X*_1_ *X*_3_	245.24	1	245.24	4.75	0.0469
*X*_1_ *X*_4_	0.3422	1	0.3422	0.0066	0.9363
*X*_2_ *X*_3_	253.46	1	253.46	4.91	0.0438
*X*_2_ *X*_4_	126.08	1	126.08	2.44	0.1404
*X*_3_ *X*_4_	317.98	1	317.98	6.16	0.0264
*X*_1_^2^	584.97	1	584.97	11.33	0.0046
*X*_2_^2^	0.4333	1	0.4333	0.0084	0.9283
*X*_3_^2^	548.65	1	548.65	10.63	0.0057
*X*_4_^2^	1388.95	1	1388.95	26.90	0.0001
Residual	722.83	14	51.63		
Lack of Fit	620.44	10	62.04	2.42	
Pure Error	102.39	4	25.60		
Total	86566.89	28			
R^2^ = 0.9917 Adjusted R^2^ = 0.9833 Predicted R^2^ = 0.9569 Adeq Precision = 41.6304					

**Table 9:** ANOVA of the regression model for top width.

Source	Sum of Squares	df	Mean Square	*F* Value	*p* Value
Model	4654.83	14	332.49	27.56	<0.0001
Laser Power (W) *X*_1_	2524.62	1	2524.62	209.23	<0.0001
Cutting Speed (mm/s) *X*_2_	729.96	1	729.96	60.50	<0.0001
Pulse Width (μs) *X*_3_	1.66	1	1.66	0.1377	0.7161
Water Pressure (MPa) *X*_4_	5.35	1	5.35	0.4437	0.5162
*X*_1_ *X*_2_	2.70	1	2.70	0.2240	0.6433
*X*_1_ *X*_3_	307.93	1	307.93	25.52	0.0002
*X*_1_ *X*_4_	1.41	1	1.41	0.1166	0.7379
*X*_2_ *X*_3_	1.59	1	1.59	0.1321	0.7217
*X*_2_ *X*_4_	21.21	1	21.21	1.76	0.2061
*X*_3_ *X*_4_	0.3956	1	0.3956	0.0328	0.8589
*X*_1_^2^	93.72	1	93.72	7.77	0.0145
*X*_2_^2^	1.99	1	1.99	0.1652	0.6906
*X*_3_^2^	803.80	1	803.80	66.62	<0.0001
*X*_4_^2^	44.87	1	44.87	3.72	0.0743
Residual	168.93	14	12.07		
Lack of Fit	129.94	10	12.99	1.33	0.4200
Pure Error	38.99	4	9.75		
Total	4823.76	28			
R^2^ = 0.9650 Adjusted R^2^ = 0.9300 Predicted R^2^ = 0.8322 Adeq Precision = 22.0022					

**Table 10:** ANOVA of the regression model for MRR.

Source	Sum of Squares	df	Mean Square	*F* Value	*p* Value
Model	469.75	14	33.55	337.43	<0.0001
Laser Power (W) *X*_1_	111.63	1	111.63	1122.58	<0.0001
Cutting Speed (mm/s) *X*_2_	328.65	1	328.65	3305.03	<0.0001
Pulse Width (μs) *X*_3_	9.90	1	9.90	99.57	<0.0001
Water Pressure (MPa) *X*_4_	0.0675	1	0.0675	0.6788	0.4238
*X*_1_ *X*_2_	7.29	1	7.29	73.31	<0.0001
*X*_1_ *X*_3_	0.1225	1	0.1225	1.23	0.2857
*X*_1_ *X*_4_	0.0025	1	0.0025	0.0251	0.8763
*X*_2_ *X*_3_	0.1225	1	0.1225	1.23	0.2857
*X*_2_ *X*_4_	0.5625	1	0.5625	5.66	0.0322
*X*_3_ *X*_4_	0.3025	1	0.3025	3.04	0.1030
*X*_1_^2^	2.42	1	2.42	24.34	0.0002
*X*_2_^2^	1.25	1	1.25	12.58	0.0032
*X*_3_^2^	4.17	1	4.17	41.92	<0.0001
*X*_4_^2^	2.35	1	2.35	23.61	0.0003
Residual	1.39	14			
Lack of Fit	0.9642	10	0.0964	0.9011	0.5957
Pure Error	0.4280	4	0.1070		
Total	471.15	28			
R^2^ = 0.9970 Adjusted R^2^ = 0.9941 Predicted R^2^ = 0.9868 Adeq Precision = 75.0136					

**Table 11:** Process parameter optimization scheme.

Serial Number	X_1_ (W)	X_2_ (mm/s)	X_3_ (μs)	X_4_ (Mpa)	D (μm)	TW (μm)	MRR (mg/s)	Desirability
1	239.774	0.900	36.912	2.143	459.999	230.000	21.816	0.988
2	238.624	0.896	36.839	2.134	460.001	230.000	21.759	0.988
3	237.461	0.900	37.093	2.108	460.000	230.000	21.702	0.987

**Table 12:** Validation test results of optimization scheme.

Serial Number	X_1_ (W)	X_2_ (mm/s)	X_3_ (μs)	X_4_ (Mpa)	D (μm)	Δ*_D_* (%)	TW (μm)	Δ*_TW_* (%)	MRR (mg/s)	Δ_MRR_ (%)
1	239.8	0.90	36.9	2.14	469.384	2.04	236.521	2.84	20.413	6.43
2	238.6	0.90	36.8	2.13	467.257	1.58	230.727	0.32	20.351	6.47
3	237.5	0.90	37.1	2.11	448.695	2.46	231.886	0.82	19.995	7.87

## Data Availability

Not applicable.

## References

[1] Li Z.Y., Liu X.L., Wu G.Q., Huang Z. (2019). Fretting fatigue behavior of Ti-6Al-4V and Ti-10V-2Fe 3Al alloys. Met. Mater. Int..

[2] Hamza H.M., Deen K.M., Haider W. (2020). Microstructural examination and corrosion behavior of selective laser melted and conventionally manufactured Ti6Al4V for dental applications. Mater. Sci. Eng. C.

[3] Liu S., Shi Y.C. (2019). Additive manufacturing of Ti6Al4V alloy: A review. Mater. Des..

[4] Froend M., Fomin F., Riekehr S., Alvarez P., Zubiri F., Bauer S., Klusemann B., Kashaev N. (2017). Fiber laser welding of dissimilar titanium (Ti-6Al-4V/cp-Ti) T-joints and their laser forming process for aircraft application. Opt. Laser Technol..

[5] Sun Z.P., He G.Y., Meng Q.J., Li Y., Tian X. (2020). Corrosion mechanism investigation of TiN/Ti coating and TC4 alloy for aircraft compressor application. Chin. J. Aeronaut..

[6] Mandlenkosi G.M., Lethu C., Peter A.O. (2022). Study of the corrosion properties of powder rolled Ti-6Al-4V alloy applied in the biomedical implants. J. Mater. Res. Technol..

[7] El-Hossary F.M., Negm N.Z., Abd El-Rahman A.M., Seleem A.A., Abd El-Moula A.A. (2015). Tribo-mechanical and electrochemical properties of plasma nitriding titanium. Surf. Coat. Technol..

[8] Ju J., Zhao C., Kang M., Li J., He L., Wang C., Li J., Fu H., Wang J. (2021). Effect of heat treatment on microstructure and tribological behavior of Ti-6Al-4V alloys fabricated by selective laser melting. Tribol. Int..

[9] Niu R., Li J., Wang Y., Chen J., Xue Q. (2016). Structure and tribological behavior of GLCH/nitride coupled coatings on Ti6Al4V by nitriding and magnetron sputtering. Diam. Relat. Mater..

[10] Wang L., Yang L., Huang Y., Yuan Y., Jia C. (2021). Effects of Y2O3 addition on the microstructure and wear-resistant performance of TiN/TiB-reinforced Ti-based laser-clad coatings on Ti-6Al-4V alloys. Mater. Today Commun..

[11] He D., Zheng S., Pu J., Zhang G., Hu L. (2015). Improving tribological properties of titanium alloys by combining laser surface texturing and diamond-like carbon film. Tribol. Int..

[12] Fatoba O.S., Adesina O.S., Popoola A.P.I. (2018). Evaluation of microstructure, microhardness, and electrochemical properties of laser-deposited Ti-Co coatings of Ti-6Al-4V alloy. Int. J. Adv. Manuf. Technol..

[13] Sibisi P.N., Popoola AP I., Kanyane L.R., Fatoba O.S., Adesina O.S., Arthur N.K., Pityana S.L. (2019). Microstructure and microhardness characterization of CpTi/SiAlON composite coatings on Ti-6Al-4V by laser cladding. Procedia Manuf..

[14] Gao Q.S., Yan H., Qin Y., Zhang P., Guo J., Chen Z., Yu Z. (2019). Laser cladding Ti-Ni/TiN/TiW + TiS/WS2 self-lubricating wear resistant composite coating on Ti-6Al-4V alloy. Opt. Laser Technol..

[15] Sun Q., Hu T., Fan H., Zhang Y., Hu L. (2016). Thermal oxidation behavior and tribological properties of textured TC4 surface: Influence of thermal oxidation temperature and time. Tribol. Int..

[16] Wang C.C., Li Z.P., Zhao H., Zhang G., Ren T., Zhang Y. (2020). Enhanced anticorrosion and antiwear properties of Ti-6Al-4V alloys with laser texture and graphene oxide coatings. Tribol. Int..

[17] Sugihara T., Singh P., Enomoto T. (2017). Development of novel cutting tools with dimple textured surfaces for dry machining of aluminum alloys. Procedia Manuf..

[18] Adeyemi K., Sun B., Xue W., Liu W., Cao Y. (2021). Friction and wear characteristics modification via laser surface textured grooves. Surf. Eng..

[19] Wang C., Hong J., Cui M., Huang H., Zhang L., Yan J. (2022). The effects of simultaneous laser nitriding and texturing on surface hardness and tribological properties of Ti6Al4V. Surf. Coat. Technol..

[20] Bonse J., Kirner S.V., Griepentrog M., Spaltmann D., Krüger J. (2018). Femtosecond laser texturing of surfaces for tribological applications. Materials.

[21] Khanh P.L., Rie T., Noboru Y., Yoshiro I. (2020). Laser-Assisted Wet Etching of Silicon Back Surfaces Using 1552nm Femtosecond Laser. Int. J. Electr. Mach..

[22] Kumar M., Melkote S.N. (2012). Process capability study of laser assisted micro milling of a hard-to-machine material. J. Manuf. Process..

[23] Feng S., Huang C., Wang J., Jia Z. (2019). Surface quality evaluation of single crystal 4H-SiC wafer machined by hybrid laser-waterjet: Comparing with laser machining. Mater. Sci. Semicond. Process..

[24] Porter J.A., Louhisalmi Y.A., Karjalainen J.A., Füger S. (2007). Cutting thin sheet metal with a water jet guided laser using various cutting distances, feed speeds and angles of incidence. Int. J. Adv. Manuf. Technol..

[25] Mulick S., Madhukar Y.K., Roy S., Nath A.K. (2016). Performance optimization of water-jet assisted underwater laser cutting of AISI 304 stainless steel sheet. Opt. Lasers Eng..

[26] Madhukar Y.K., Mullick S., Nath A.K. (2016). A study on co-axial water-jet assisted fiber laser grooving of silicon. J. Mater. Process. Technol..

[27] Adelmann B., Ngo C., Hellmann R. (2015). High aspect ratio cutting of metals using water jet guided laser. Int. J. Adv. Manuf. Technol..

[28] Sun D., Han F., Ying W. (2019). The experimental investigation of water jet-guided laser cutting of CFRP. Int. J. Adv. Manuf. Technol..

[29] Tohidifar M.R. (2017). On the analysis and optimization of lithi um-mica nano-crystallites using a statistical technique. Mater. Charact..

[30] Loutas T.H., Kliafa P.M., Sotiriadis G., Kostopoulos V. (2019). Investigation of the effect of green laser pre-treatment of aluminum alloys through a design-of-experiments approach. Surf. Coat. Technol..

[31] Zhao W., Ma A., Ji J., Chen X., Yao T. (2020). Multiobjective Optimization of a Double-Side Linear Vernier PM Motor Using Response Surface Method and Differential Evolution. IEEE Trans. Ind. Electron..

[32] Xing Y., Liu L., Hao X., Wu Z., Huang P., Wang X. (2018). Micro-channels machining on polycrystalline diamond by nanosecond laser. Opt. Laser Technol..

[33] Mohammec B.S., Khed V.C., Nuruddin M.F. (2018). Rubbercrete mixture optimization using response surface methodology. J. Clean. Prod..

[34] Liu Q., Zhao Y., Meng J., Zhao G., Zhou H., Li L., Wang K., Liu G., Cao C., Zheng Z. (2022). Effect of static alignment deviation on coupling efficiency and beam quality of water beam fiber. Appl. Opt..

[35] Liu Q., Zhao Y., Meng J., Zhao G., Li L., Zhou H., Wang K., Liu G., Cao C., Zheng Z. (2022). Research on the influence of water-laser coupling cavity and nozzle structure on the flow characteristics of water beam fiber. Iran. J. Sci. Technol. Trans. Mech. Eng..

